# Metallization of Recycled Glass Fiber-Reinforced Polymers Processed by UV-Assisted 3D Printing

**DOI:** 10.3390/ma15186242

**Published:** 2022-09-08

**Authors:** Alessia Romani, Paolo Tralli, Marinella Levi, Stefano Turri, Raffaella Suriano

**Affiliations:** 1 Department of Chemistry, Materials and Chemical Engineering “Giulio Natta”, Politecnico di Milano, Piazza Leonardo da Vinci 32, 20133 Milano, Italy; 2 Design Department, Politecnico di Milano, Via Durando 10, 20158 Milano, Italy; 3 Divisione Green Coat, Novellini S.p.a., Strada Romana Nord, 1, 46027 San Benedetto Po, Italy

**Keywords:** additive manufacturing, 3D printing, surface finishing, physical vapor deposition, composites, recycling, polymer-matrix composites (PMCs), direct ink writing, liquid deposition modeling, glass fibers

## Abstract

An ever-growing amount of composite waste will be generated in the upcoming years. New circular strategies based on 3D printing technologies are emerging as potential solutions although 3D-printed products made of recycled composites may require post-processing. Metallization represents a viable way to foster their exploitation for new applications. This paper shows the use of physical vapor deposition sputtering for the metallization of recycled glass fiber-reinforced polymers processed by UV-assisted 3D printing. Different batches of 3D-printed samples were produced, post-processed, and coated with a chromium metallization layer to compare the results before and after the metallization process and to evaluate the quality of the finishing from a qualitative and quantitative point of view. The analysis was conducted by measuring the surface gloss and roughness, analyzing the coating morphology and thickness through the Scanning Electron Microscopy (SEM) micrographs of the cross-sections, and assessing its adhesion with cross-cut tests. The metallization was successfully performed on the different 3D-printed samples, achieving a good homogeneity of the coating surface. Despite the influence of the staircase effect, these results may foster the investigation of new fields of application, as well as the use of different polymer-based composites from end-of-life products, i.e., carbon fiber-reinforced polymers.

## 1. Introduction

Polymer-based composites are increasingly employed for a large number of applications ranging from aerospace and automotive to healthcare and construction [1]. Their extensive use in different sectors mainly derives from their versatility and technical properties, i.e., high strength-to-weight ratio. Although the COVID-19 pandemic strongly impacted the composites market, the production volumes of fiber-reinforced polymers are reaching the pre-crisis levels of 2019, especially considering short fiber-reinforced materials, i.e., glass fiber-reinforced polymers (GFRPs) [2]. Consequently, this ever-increasing production will result in higher volumes of GFRP waste to be correctly managed in the coming years [3,4]. For instance, the wind energy sector will generate a growing amount of GFRPs at their end-of-life (EoL) since the lifetime of wind turbine blades usually corresponds to approximately 20–25 years, leading to the disposal of an ever-growing number of windfarms [5]. Despite the current challenges in reusing and recycling composite materials [6,7], new solutions have been investigated to reintroduce GFRP waste in new closed- and open-loop systems, following the principles of circular economy [8,9].

Among the available technologies, 3D printing, or Additive Manufacturing (AM) represents a promising way to reprocess recycled GFRPs thanks to its flexibility in terms of material formulations and customizable complex geometries [10,11]. From the literature, different formulations of GFRPs and carbon fiber-reinforced polymers (CFRPs) were 3D-printed through FDM (fused deposition modeling, or FFF, fused filament fabrication) and DIW (direct ink writing, or LDM, liquid deposition modeling), showing their use for a wide range of applications [12,13,14,15]. Recently, some works have also demonstrated their capability to foster this sustainable transition within the composite market by reprocessing recycled GFRPs and CFRPs [16,17,18,19,20,21]. Nevertheless, the surface properties of these 3D-printed recycled composites may considerably differ from pristine materials, as well as their aesthetic appearance. Furthermore, some applications require different technical properties, which can be achieved only through a post-processing treatment.

Metallization represents an effective way to combine the advantages of polymer-based composites with those of metallic surfaces. Metallization coatings can be deposited through different chemical or physical techniques such as electroless plating or thermal spray deposition [22]. In particular, physical vapor deposition (PVD) sputtering stands out for its coating homogeneity, low processing temperatures, and the use of less dangerous chemicals during the metal deposition [23,24,25,26]. From the literature, different studies explored the deposition of metallic coatings onto 3D-printed thermoplastic substrates processed by FFF [27,28,29,30,31,32,33,34], whereas a limited number of works focused on the metallization of polymer-based composites [35], or parts obtained by DIW [36]. To the best of the authors’ knowledge, no articles related to the metallization of 3D-printed recycled composites were found by using neither FFF nor DIW processes. Indeed, a hard chromium coating would significantly influence not only their mechanical, surface, and chemical properties but also the overall perception of the final part by end users [37,38].

This paper aims to show the successful use of PVD sputtering for the metallization of 3D-printed GFRPs from recycled composites. First, four batches of samples made of thermoset acrylic resin filled with mechanically-recycled GFRPs from EoL wind turbine blades were 3D-printed through UV-assisted three-dimensional (UV-3D) printing. These samples were post-processed and coated with a PVD chromium sputtering deposition process. The surfaces of these samples were then assessed from a qualitative and quantitative point of view, i.e., by evaluating the aesthetic appearance, surface properties, and morphology of the coating. Considering the promising results, this work may influence further research activities focused on the post-processing of additive manufactured parts for new potential applications, especially considering emerging 3D printing processes or new polymer-based materials from composite waste and parts at their EoL.

## 2. Materials and Methods

### 2.1. Materials and UV-3D Printing

The 3D printable ink consisted of a photo- and thermo-curable acrylic matrix mixed with mechanically recycled GFRPs for 20 min at 40 rpm through a Brabender mixer equipped with a roller blade (C.W. Brabender Instruments Inc., South Hackensack, NJ, US). The matrix is composed of 96.7 wt% of ethoxylate bisphenol A diacrylate resin named SR349 (Arkema, Colombes, France and locally distributed by Came S.r.l., Lainate, Italy), 3 wt% of ethyl phenyl (2,4,6-trimethyl benzoyl) phosphinate named TPO-L (Lambson Limited, Wetherby, UK), and 0.3 wt% of dicumyl peroxide (Sigma-Aldrich Corporation, St. Louis, MO, USA). The matrix was previously mixed with a magnetic stirrer at room temperature for 2 h. The ink formulation used in this work contains 55 wt% of shredded GFRPs from EoL wind turbine blades made of continuous epoxy-based GFRPs (Siemens Gamesa Renewable Energy S.A., Zamudio, Spain, and Consiglio Nazionale di Ricerca—Sistemi e Tecnologie Industriali Intelligenti per il Manifatturiero Avanzato—Stiima CNR, Milano, Italy) and 45 wt% of the acrylic matrix. The shredded recycled GFRPs have a glass fiber content of 70 wt% and a nominal granulometry of 80 μm (34 and 14 μm of average length and diameter) [17].

The 3D samples for the surface characterization and metallization assessment have a nominal overall dimension of 50 × 25 × 30 mm and were designed with Fusion 360 CAD software (Autodesk, San Rafael, CA, USA). The UV-3D printing was performed with a custom version of a 3Drag, a commercial FFF low-cost 3D printer (Futura Group S.r.l., Gallarate, Italy). The 3D printer was converted into a UV-assisted DIW apparatus by adding a UV LED source, a 20 mL syringe, and a stainless-steel UV-shielded conic nozzle (Techcon Systems Inc, Cypress, CA, USA) [17]. The G-code file of the 3D sample was prepared with the slicing software Prusa Slicer (Prusa Research, Prague, Czech Republic), and the main 3D printing parameters are shown in Table 1. The 3D-printed samples were then post-cured to completely crosslink the acrylic-based resin. The post-curing treatment was performed in a UV chamber Polymer 500 W with a UVA emittance mercury vapor lamp for 30 min (Helios Italquartz S.r.l., Cambiago, Italy) and in a non-controlled atmosphere oven for 2 h at 140 °C.

### 2.2. PVD Sputtering and Surface Characterization

Four different batches of minimum two samples were then produced to assess the influence of 3D printing, post-processing, and chromium metallization on the surfaces, as better explained in Section 3.1. The PVD sputtering deposition was carried out by Novellini S.p.a.—Divisione Green Coat with a proprietary process (San Benedetto Po, Italy), following the same procedure used for the metallization of FFF 3D-printed samples [35]. After cleaning the surfaces of the samples with a cloth moistened with isopropyl alcohol, a UV-curable acrylic-based primer named UNILAC UV BC 05 (Cromogenia Units S.A., Barcelona, Spain) was sprayed with an average thickness of 70–80 μm to improve the surface roughness of the 3D printing process. The primer deposition was performed with an anthropomorphic robot equipped with rotary atomizer (cup spinning at 25,000 rpm) once the samples were fixed on a rotational jig. The UV-curable primer was then photo-crosslinked with 26 mercury vapor lamps with a peak irradiance of 500 mW/cm^2^ and a 10″ bulb for 2 min. The jig was moved into the in-line metallization system for the surface activation with the oxygen plasma for 90 s before the chromium deposition (O_2_ flow of 700 sccm, electrode power of 4 kW, rotation speed of 5 rpm). Afterward, the PVD magnetron sputtering was carried out to deposit a chromium layer with a nominal thickness of 200 nm. The process was performed for 16 min at a pressure of 1–5 × 10^−3^ mbar and a constant argon flow of 600 sccm. A maximum temperature of 60 °C was reached during the PVD sputtering process.

Gloss was evaluated with a Multi-gloss 268A gloss meter (Konica Minolta, Tokyo, Japan). The measurement results, expressed in Gloss Units (GU), were carried out on the planar surfaces of the samples according to ASTM D523-14 Standard “Standard Test Method for Specular Gloss” [39]. A 60° measuring geometry, or the angle of incidence of 60°, was used for the different batches to compare the gloss values and to determine when using the 20° and 85° geometries. The 20° geometry was used for gloss values higher than 70 GU, whereas the latter was for values lower than 70 GU. The measurements on each sample were repeated five times to calculate the average values and standard deviations.

The surface roughness of the 3D samples was measured with a laser profilometer (UBM) along five different lines for each surface with a point density of 150 points/mm. The values were then used to calculate the average experimental values and standard deviation of roughness average (Ra) and root mean square roughness (Rq).

The cross-section analysis of the average coating thicknesses and morphology was performed through Scanning Electron Microscopy (SEM) micrographs with an extended-pressure SEM Zeiss EVO 50 EP (Carl Zeiss S.p.A., Milano, Italy). Two different cross-sections were obtained from each sample. The surfaces of the samples were then prepared for observation through PVD of a thin gold layer. The samples were observed at 500 and 3k × magnification in high-vacuum conditions and an acceleration voltage of 10–20 kV.

Manual cross-cut tests were carried out on the planar surfaces of a batch of samples to evaluate the adhesion properties according to ISO 2409:2020 Standard “Paints and varnishes—Cross-cut test” [40]. The results were then expressed from 0 to 5, where 0 means no removals and 5 means that more than 65% of the cross-cut area is affected by detachments.

## 3. Results and Discussion

### 3.1. Three-Dimensional Printing and Metallization of the Samples

A specific 3D sample was designed to compare the influence of post-processing and PVD sputtering on the 3D-printed surfaces of the recycled GFRPs. Since UV-DIW may be considered an emerging 3D printing process, a simple geometry was defined to reduce the presence of defects due to DIW, such as missing layers for nozzle clogging [17,41]. The shape of the 3D sample was defined according to the PVD sputtering setup, whereas the overall dimensions were modified to control the printing times of each sample. In particular, the sample had a wider planar surface on the bottom to perform the main surface characterizations, i.e., gloss measurements, roughness evaluations, and cross-cut tests. The smaller planar surface, the curved profile, and the internal cavity can be used to evaluate the homogeneity of the chromium deposition on complex profiles by simulating complex features to be reached with the PVD sputtering.

To this end, four different batches were 3D printed. Each batch was composed of two samples at least and was obtained starting from the same G-code file. The batches are different in terms of post-processing and/or presence of the chromium metallization, as listed in Table 2. Different surface characterization tests were performed on each batch. Gloss measurements and roughness evaluations were carried out for each batch, whereas adhesion tests and SEM micrographs were performed on the most representative one, batch n. 2. This batch was not post-processed before the PVD sputtering; hence it should represent the most influenced batch from the UV-3D printing process and the recycled GFRP since the chromium deposition was performed on a surface with the typical layer-by-layer appearance, also known as the staircase effect.

**Figure 1:**
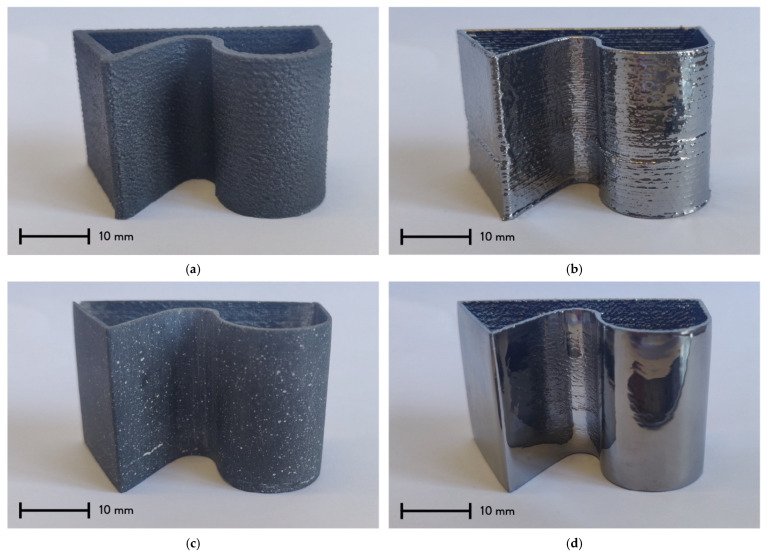
3D-printed and metallized specimens from the different sample batches: (**a**) Batch n. 1 (no finishing, no PVD); (**b**) Batch n. 2 (No finishing, PVD); (**c**) Batch n. 3 (Finishing, no PVD); (**d**) Batch n. 4 (Finishing, PVD).

The most representative 3D sample of each batch is visible in Figure 1. In general, the samples do not show defects due to the metallization (Figure 1b,d), and no detachments or unprocessed areas are visible on the chromium surface, confirming a good homogeneity of the coating deposition (Figure 1b). Furthermore, no geometrical deformations can be detected from the metallized batches (n. 2 and n. 4). Figure 1a,c represent the samples before and after the sanding post-processing. The influence of the recycled GFRP reinforcement is clearly visible in both batches. In the first case, the surface texture of the composite material slightly reduces the staircase effect of the 3D printing process. In the second case, a random clear pattern is visible on the surface, and it derives from the sanding post-processing of the composite material, highlighting the glass fiber particles of the reinforcement. Figure 1b,d show the difference in the PVD sputtering chromium layer on the samples before and after the sanding post-processing. Chromium deposition significantly changes the appearance of the 3D-printed surface by giving to the samples a glossy surface. As evidenced by the difference between Figure 1a and 1b, metallization slightly reduces the staircase effect of 3D printing. Combining sanding post-processing and PVD sputtering allows one to obtain mirror-like surfaces, widening the range of potential applications (Figure 1c,d).

### 3.2. Roughness

The roughness of the four batches of samples was measured, comparing the values not only before and after the sanding post-processing but also before and after the PVD sputtering process. The larger vertical planar surface of each sample was used for the measurements since non-sanded batches would be affected by the typical staircase effect of UV-3D printing and recycled GFRPs. Therefore, the comparisons would show the reductions in roughness linked to the sanding post-processing and the chromium deposition [37,42].

Table 3 shows the values of roughness average (Ra) and root mean square roughness (Rq) of the different batches. The lower results were reached by batch n. 4, which represents the post-processed and coated batch of samples. The overall average reduction in terms of Ra and Rq from batch n. 1 to batch n. 4 is equal to 18.22 and 22.61 μm, which means more than 99.5% in both cases. The PVD sputtering of chromium onto a non-sanded rough 3D-printed substrate (batch n. 2) halved the Ra and Rq values, while a fifty-fold decrease was observed by carrying out the PVD sputtering onto a sanded substrate (batch n. 4). Furthermore, the values show good accuracy of the measurements considering the low standard deviations, especially for the PVD sputtering batches, confirming the homogeneity of the chromium coatings. These values were also obtained thanks to the primer deposition, which improved the roughness homogeneity of the substrate by reducing the peaks and valleys of the 3D-printed surface.

### 3.3. SEM Analysis

**Figure 2:**
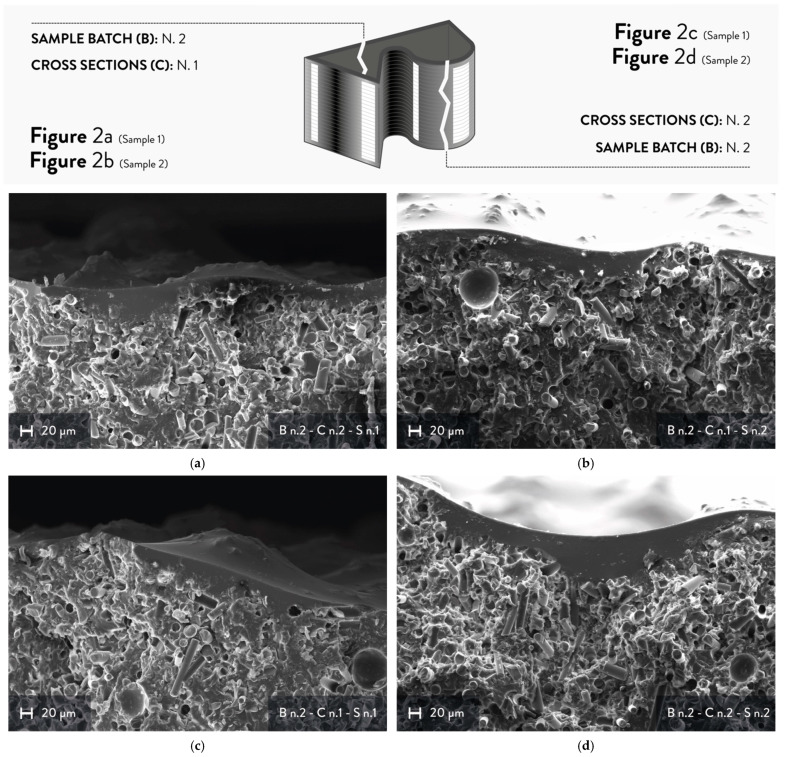
SEM micrographs of cross-sections from batch n. 2 (500× magnification): (**a**) cross-section n. 1, sample n. 1; (**b**) cross-section n. 1, sample n. 2; (**c**) cross-section n.2, sample n. 1; (**d**) cross-section n. 2, sample n. 2.

The cross-sections were analyzed through SEM micrographs to determine the average thickness of the PVD sputtering coating and assess its morphology. Two different cross-sections were evaluated for each sample of batch n. 2 (Figure 2). According to the results from the roughness measurements (Table 3), this batch allows better observation of the influence of the staircase effect on the PVD sputtering coating because of its higher Ra, Rq, and corresponding standard deviations compared to the sanded sputtered sample.

As shown in Figure 2, the PVD sputtering coating is quite visible because of the evident difference between the recycled GFRP substrate and the UV primer. The former can be detected for the presence of fibers, whereas the latter corresponds to the less irregular area. However, the interface between the substrate and the coating is not clearly distinguishable, giving only an idea of the general contours of the 3D-printed substrate. This aspect is probably due to the composition of the UV primer, which is similar to the acrylic-based matrix of the 3D printable ink. For this reason, the adhesion between the substrate and the UV primer may be increased, although this property of the PVD sputtering coating is strongly affected also by the surface roughness of the sample.

**Figure 3:**
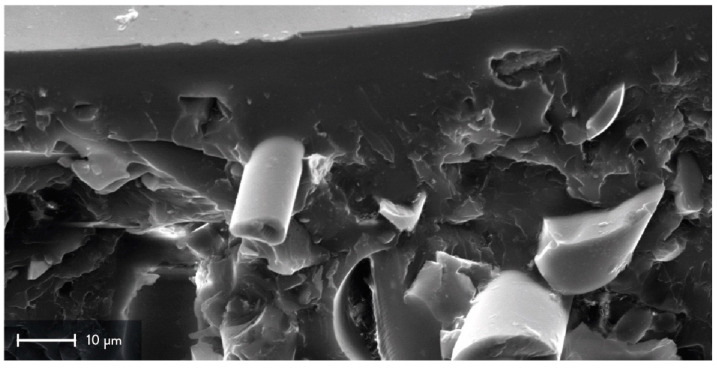
SEM micrographs of a representative cross-section from batch n. 2: insight on the fiber adhesion with the PVD sputtering coating and the UV primer (3k× magnification) highlighted from the cylindrical structure in the middle, which shows a recycled glass fiber.

The chromium layer is not easily visible because of its low contrast with the primer and the substrate, as shown in Figure 3. Hence, its average thickness was not measured nor compared with its nominal value of 200 nm. Indeed, the average thickness of the whole PVD sputtering coating was evaluated, which means the chromium and the UV primer layers. The latter was sprayed onto the surface before the PVD sputtering process to improve the adhesion of the Cr layer, and the nominal value of its thickness is equal to 60–70 μm. The measurements were carried out during the SEM analysis by measuring three points of at least two micrographs for each cross-section. The experimental average thickness of the PVD sputtering coating is equal to 37.42 ± 21.04 μm. Compared to the nominal thickness, this value is lower than expected, and it could be justified by the Ra and Rq values of batch n. 2. After the spray deposition, the UV primer smoothed the surface of the 3D-printed substrate by filling the valleys, resulting in a less homogeneous layer. However, the non-homogeneous thickness of the coating led to lower values of roughness, confirming the overall quality of the PVD sputtering observed during the first qualitative analysis (Section 3.1). Figure 2a,b show the cross-sections of the first specimen of NF_PVD, whereas the cross-sections of the second sample are visible in Figure 2c,d.

The analysis of the cross-sections at higher magnification shows a good adhesion between the UV primer and the recycled glass fibers, represented by the cylindrical structures of Figure 3. Considering the substrate is made of recycled GFRPs, some fibers may be directly exposed to the external surface. As for other composite substrates, this issue may strongly affect the overall quality of the coating since the reinforcement often has different compositions and properties compared to the matrix. In this case, good adhesion of the UV primer may increase instead the homogeneity of the substrate, improving the surface properties of the final coating.

### 3.4. Gloss Measurements

After the qualitative assessment of the 3D-printed surfaces appearance in Section 3.1, gloss measurements were performed to define a quantitative value for each batch of samples. Table 4 shows the results from the gloss evaluation. According to ASTM D523-14 [39], the first measurements were performed with the 60° geometry, allowing a general comparison between the four batches. The samples before and after the PVD sputtering show different values, resulting in an increase in gloss after the metallization, from 2–4 GU to 113–130 GU. In general, values higher than 70 GU at 60° refer to high-gloss surfaces, whereas values lower than 10 GU indicate surfaces with low gloss. Gloss measurements for the metallized samples showed higher values of standard deviations. This was probably due to the standard deviation values measured for surface roughness that were of the same order of magnitude of Ra and Rq average values for samples after PVD sputtering (Section 3.2). As a matter of fact, the staircase effect influences gloss values since it contributes to increasing surface roughness values. Compared to smooth surfaces, i.e., the sanded 3D samples, the staircase effect also changes the specular reflectivity of the surface, leading to higher variability of the gloss measurements [37].

Samples before the PVD sputtering from batches n. 1 and n. 3 were then evaluated with the 85° geometry to have more accurate measures. In this case, the gloss value increases for the sample from batch n. 3, which means after the sanding post-processing. The result is in line with the decrease in surface roughness. Batches n. 2 and n. 4 were finally evaluated with the 20° geometry for high gloss surfaces, improving the accuracy of the measurements. These results, together with the roughness measurements, confirm the qualitative assessment of the 3D samples shown in the previous Section 3.1, highlighting the improvement of surface properties for sanded and Cr-sputtered samples, resulting in mirror-like 3D-printed surfaces.

### 3.5. Adhesion Properties

Manual cross-cut tests were performed on the samples of batch n. 2 to assess the adhesion level of the chromium coating. As mentioned before, the metallized surfaces of this batch are the most affected by the staircase effect of the 3D-printed samples. Therefore, to assess the influence of 3D-printed recycled composites on adhesion, the cross-cut tests were performed only for batch n.2. According to ISO 2409:2020 [40], the adhesion level between the chromium layer and the 3D-printed substrate is equal to 2. In detail, the coating has slightly flacked along the edges of the cross-cut squares, and detachment affected an area between 5 and 15% of the tested surface. This result is significantly affected by the staircase effect that generally shows worse values compared to smooth surfaces, decreasing the adhesion properties [34,43].

### 3.6. Fields of Application

After the qualitative analysis and surface characterization, some considerations can be made about the possible application fields. Although these coatings were initially used in the electronic and medical sectors [44,45,46], PVD sputtering on 3D-printed parts may be employed for a larger number of applications since it improves the properties of the parts, i.e., wear resistance, durability, and surface aesthetics [30,47]. Moreover, a coating process can also reduce the effects of the 3D printing process such as the staircase effect, which may influence not only the appearance of a 3D printed product but also its functional properties.

The deposition of metallic layers onto 3D-printed thermoplastic parts has been studied very recently, confirming the increasing interest in its future industrial exploitation [26,31,34,35]. Similarly, using PVD sputtering processes for polymer-based composites may generate potential benefits, especially for recycled substrates. Focusing on the case study of this paper, high-performance applications could be exploited by using coated recycled GFRPs, such as technical parts, outdoor furniture, or customized scenery elements [17,48]. In detail, these kinds of recycled GFRPs exhibit interesting mechanical properties for similar applications, showing good values of elastic modulus (~5.5–6 GPa) and toughness (~200 J/mm^3^), which can be comparable to virgin GFRPs [17]. Considering the suitability of these materials for similar applications, the PVD sputtering coating may further enhance their durability by acting as a protective film for the 3D-printed substrate. Moreover, this coating could reduce or eliminate the superficial defects of the DIW process such as voids, which could be a cause for the failure of the parts themselves. Considering the 3D printing process, this coating may also limit the delamination caused by low intralayer adhesion thanks to the use of the acrylic UV primer, which showed good compatibility with the acrylic-based matrix of the 3D printable ink.

PVD sputtering can also improve the homogeneity of the surface of parts made with recycled GFRPs. First, the surface of composite materials often shows local variations in the composition in correspondence to the matrix or the reinforcement, leading to non-homogeneous surface properties. The different batches of recycled glass fibers can significantly change from a morphological point of view, i.e., average granulometry, affecting the technical properties and the overall aesthetics.

According to the previous assumptions, PVD sputtering for 3D-printed recycled composites could be proposed for the automotive and furniture sectors. For the former, metallized 3D-printed recycled composites may be used for high-performing and customized batches of small- and medium-format parts, whereas for the latter it shows good potential for large-format customized products [49,50]. As a matter of fact, this coating technology influences not only the technical properties of a 3D-printed product but also its expressive-sensorial qualities, influencing the perception of customers and end-users [35,51]. In this case, the aesthetics of recycled GFRPs may be a desired feature or not, according to the specific designer’s choice and the users’ target. However, metallization can add several advantages to the final product made of recycled GFRPs by considering the principles of Design for Sustainability [52,53], i.e., improving the durability of the parts, extending the life cycle of the product, and fostering the acceptance of recycled materials amongst the end-users. Further studies are necessary to check the durability of the 3D-printed GFRP samples coated with a chromium metallization layer. However, some promising pieces of evidence were presented in the literature, showing the increased resistance to water droplet erosion of various GFRPs electro-deposited with metal layers, e.g., Cr on Cu, when compared to samples without metallic coatings [54].

## 4. Conclusions

This work aimed to demonstrate the feasibility of PVD sputtering for the metallization of 3D-printed polymer-based composites from EoL products. Four batches of samples made of thermoset acrylic resin filled with mechanically recycled GFRPs were fabricated through UV-assisted 3D printing and coated by means of chromium PVD sputtering. From the qualitative assessment of these samples, no visible defects, unprocessed areas, or detachments were detected, showing a good homogeneity of the coating. No geometrical deformations were found, and the staircase effect of the sample was mitigated by the PVD sputtering process. A reduction in the roughness values of more than 99.5% was observed after PVD sputtering, whereas performing the metallization on a sanded batch led to a fifty-fold decrease in these values. The SEM analysis of the cross section showed the influence of the UV primer deposition in smoothing the surface of the substrate, obtaining a more homogeneous coating by filling the valleys derived from the staircase effect. However, the adhesion properties of the metallized coatings were lower than expected, and this issue probably derives from the staircase effect, which strongly influences the roughness of non-sanded samples. Carrying out the cross-cut test on smooth sanded and coated surfaces, which showed a less evident staircase effect, should improve this result. The surface gloss was then evaluated, showing a general increase in gloss after the metallization. However, the staircase effect influenced the measurements leading to higher variability.

According to these results, PVD sputtering onto 3D-printed recycled GFRPs can be exploited for new fields of applications, such as automotive and furniture. This metallization treatment can influence the technical properties and the aesthetics of new 3D-printed products made of recycled composites, allowing industries, designers, and engineers to foster the use of these kinds of materials. Future work should be done to deepen the surface characterization of this surface treatment for DIW, i.e., through more complex geometries. Furthermore, other polymer-based composites could be used, such as virgin and recycled carbon fibers. In this way, additional high-performance sectors will benefit from these promising results, i.e., the aerospace field.

## Figures and Tables

**Table 1:** Three-dimensional printing parameters of the samples for the surface characterization.

Parameters	Units	Values
Perimeters	-	1
Infill	%	0
Flow	%	100
Speed	mm/s	15
Layer height	mm	0.25
Nozzle diameter	mm	1
UV LED source	-	3 × 3 W (395 nm)

**Table 2:** Sample batches with the corresponding specifics (post-processing, metallization) and characterization tests.

Sample (Batch)	Figure	Post-Processing	Metallization	Tests
N. 1	Figure 1a	No;	No;	Gloss, Roughness;
N. 2	Figure 1b	No;	Yes (sanding), PVD sputtering;	Gloss, Roughness, SEM, Adhesion;
N. 3	Figure 1c	Yes, sanding;	No;	Gloss, Roughness;
N. 4	Figure 1d	Yes, sanding;	Yes (sanding), PVD sputtering;	Gloss, Roughness;

**Table 3:** Vertical surface roughness average (Ra) and root mean square roughness (Rq) of the sample batches.

Sample (Batch)	Ra (μm)	Rq (μm)
N. 1	18.25 ± 2.06	22.66 ± 1.75
N. 2	9.51 ± 1.39	12.25 ± 2.00
N. 3	1.60 ± 0.33	2.58 ± 0.67
N. 4	0.03 ± 0.01	0.05 ± 0.01

**Table 4:** Gloss values of the sample batches at different angles (20°, 60°, and 85°) according to ASTM D523-14 (Standard Test Method for Specular Gloss) [39].

Sample (Batch)	Standard	GU at 20°	GU at 60°	GU at 85°
N. 1	Figure 1a	//	2.34 ± 0.55	2.52 ± 1.65
N. 2	Figure 1b	39.20 ± 8.81	130.16 ± 15.82	//
N. 3	Figure 1c	//	3.76 ± 1.25	12.44 ± 0.93
N. 4	Figure 1d	15.96 ± 4.85	113.94 ± 30.88	//

## Data Availability

Publicly available data sets were analyzed in this study. The data can be found here: [https://github.com/piuLAB-official/Dataset_A.Romani_2022_Materials] (accessed on 28 July 2022). If these data are used, please cite them in the following way: [data set] Alessia Romani, Raffaella Suriano, Paolo Tralli, Marinella Levi, and Stefano Turri. 2022. Metallization of Recycled Glass Fiber Reinforced Polymers 3D-printed by UV-Assisted Direct Ink Writing; https://github.com/piuLAB-official/Dataset_A.Romani_2022_Materials (accessed on 28 July 2022).
